# Agonist-specific desensitization of PGE_2_-stimulated cAMP signaling due to upregulated phosphodiesterase expression in human lung fibroblasts

**DOI:** 10.1007/s00210-019-01800-5

**Published:** 2019-12-28

**Authors:** Francisco J. Nunez, Nancy A. Schulte, David M. Fogel, Joel Michalski, Stephen I. Rennard, Raymond B. Penn, Myron L. Toews, Rennolds S Ostrom

**Affiliations:** 1 Department of Biomedical and Pharmaceutical Sciences, Chapman University School of Pharmacy, Irvine, CA 92672, USA; 2 Department of Pharmacology and Experimental Neuroscience, University of Nebraska Medical Center, Omaha, NE, USA; 3 Department of Internal Medicine, Section of Critical Care and Sleep Medicine, University of Nebraska Medical Center, Omaha, NE, USA; 4 Division of Pulmonary and Critical Care Medicine, Department of Medicine, Center for Translational Medicine, The Jane & Leonard Korman Respiratory Institute, Thomas Jefferson University, Philadelphia, PA, USA

**Keywords:** PGE_2_, Pulmonary fibrosis, Phosphodiesterase, cAMP, EP_2_ receptors

## Abstract

Pulmonary fibrosis is characterized by fibroblasts persisting in an activated form, producing excessive fibrous material that destroys alveolar structure. The second messenger molecule cyclic 3′,5′-adenosine monophosphate (cAMP) has antifibrotic properties, and prostaglandin E_2_ (PGE_2_) can stimulate cAMP production through prostaglandin E (EP)_2_ and EP_4_ receptors. Although EP receptors are attractive therapeutic targets, the effects of long-term exposure to PGE_2_ have not been characterized. To determine the effects of long-term exposure of lung fibroblasts to PGE_2_, human fetal lung (HFL)-1 cells were treated for 24 h with 100 nM PGE_2_ or other cAMP-elevating agents. cAMP levels stimulated by acute exposure to PGE_2_ were measured using a fluorescent biosensor. Pretreatment for 24 h with PGE_2_ shifted the concentration-response curve to PGE_2_ rightward by approximately 22-fold but did not affect responses to the beta-adrenoceptor agonist isoproterenol. Neither isoproterenol nor forskolin pretreatment altered PGE_2_ responses, implying that other cAMP-elevating agents do not induce desensitization. Use of EP_2_- and EP_4_-selective agonists and antagonists suggested that PGE_2_-stimulated cAMP responses in HFL-1 cells are mediated by EP_2_ receptors. EP_2_ receptors are resistant to classical mechanisms of agonist-specific receptor desensitization, so we hypothesized that increased PDE activity mediates the loss of signaling after PGE_2_ pretreatment. PGE_2_ treatment upregulated messenger RNA for PDE3A, PDE3B, PDE4B, and PDE4D and increased overall PDE activity. The PDE4 inhibitor rolipram partially reversed PGE_2_- mediated desensitization and PDE4 activity was increased, but rolipram did not alter responses to isoproterenol. The PDE3 inhibitor cilostazol had minimal effect. These results show that long-term exposure to PGE_2_ causes agonist-specific desensitization of EP_2_ receptor-stimulated cAMP signaling through the increased expression of PDE isozymes, most likely of the PDE4 family.

## Introduction

Pulmonary fibrosis (PF) is characterized by continuous scarring of the lungs (Dackor et al. 2011). Previously, PF was thought to result from an inflammatory response (Liu et al. 2004). However, anti-inflammatory treatments show little to no effect in slowing the progression of PF and clinical out-comes were not improved (Ostrom 2014). Current understanding of PF has shifted from it being an inflammatory process to a focus on tissue damage with defects in subsequent healing and repair processes and the cellular signaling pathways that regulate them (Liu et al. 2004).

The second messenger molecule cyclic 3′,5′-adenosine monophosphate (cAMP) will (1) slow fibroblast migration to the site of injury, (2) prevent differentiation of fibroblasts into myofibroblasts, (3) reduce the amount of collagen 1*α*(II) and 5*α*(I), and (4) increase the activity of certain matrix metalloproteinases, which are responsible for degrading ECM proteins (Yamaguchi et al. 1988; Kohyama et al. 2002a; Kolodsick et al. 2003; Liu et al. 2004). Thus, cAMP is an antifibrotic second messenger. cAMP is generated in the cell when certain agonists bind to their cognate G protein–coupled receptors (GPCRs) to activate the G protein G*α*s, which, in turn, stimulates adenylyl cyclase (AC) (Liu et al. 2004). Once activated, AC converts adenosine triphosphate (ATP) into cAMP, which subsequently binds to protein kinase A (PKA) and the exchange protein activated by cAMP (Epac) (Liu et al. 2010; Insel et al. 2012). This, in turn, leads to regulation of a myriad of different proteins and pathways to produce different downstream responses (Liu et al. 2010). cAMP signaling is terminated by phosphodiesterase (PDE) enzymes that degrade cAMP and halt the signaling cascade (Liu et al. 2004). Although G*α* has several different isoforms, only G*α*s is responsible for stimulating cAMP production in the cell, and hence, the G*α*^S^-AC-cAMP pathway is pertinent to the study of cAMP and its antifibrotic effects (Liu et al. 2004).

Elevating cAMP levels for 24 h via treatment with forskolin, isoproterenol, prostaglandin E_2_ (PGE_2_), butaprost, or beraprost, or by overexpression of adenylyl cyclase 6 (AC6), inhibits fibroblast proliferation and collagen synthesis (Liu et al. 2004, 2008, 2010). Moreover, increasing cAMP levels over 24 h lowers messenger RNA (mRNA) levels of collagen types 1*α*(II) and 5*α*(I) (Liu et al. 2004). Exposure to these cAMP-elevating agents for 24 h also increases expression of matrix metalloproteinase-2 (Liu et al. 2004). PGE_2_, acting via prostaglandin E (EP)_2_ receptors stimulating cAMP in lung fibroblasts, disrupts calcium signaling and induces an antifibrotic phenotype (Mukherjee et al. 2019). Activation of cAMP response element–binding (CREB) protein via phosphorylation inhibits the profibrotic TGF-β signaling pathway by inhibiting SMAD-mediated transcriptional activation of fibrotic genes (Liu et al. 2005). cAMP elevation by prostacyclin receptors inhibits the transcription cofactors YAP/TAZ to oppose TGF-β–stimulated fibrotic responses (Zmajkovicova et al. 2019). Thus, cAMP induces diverse antifibrotic effects and approaches for increasing cAMP levels should be considered for PF therapy.

PGE_2_ is associated with inflammation and with repair of tissue injury (Liu et al. 2004). PGE_2_ has antifibrotic properties due to activation of the G*α*s-AC-cAMP pathway (Liu et al. 2004). PGE_2_ activates all EP receptors, of which EP_2_ and EP_4_ isoforms couple to the G*α*s-AC-cAMP pathway (Bozyk and Moore 2011). EP receptor subtypes EP_2_ and EP_4_ are involved in antifibrotic activity in fibroblast cells from various organs. Given the therapeutic potential of targeting these receptors for PF, understanding the effects of long-term exposure of receptors to PGE_2_ represents a novel and important basic research question. Furthermore, PF fibroblast cells are refractory to PGE_2_ receptor responses (Bozyk and Moore 2011), presumably diminishing the antifibrotic action of PGE_2_. Fibroblasts isolated from patients with chronic obstructive pulmonary disease also have altered responses to PGE_2_ as compared to those from normal subjects (Michalski et al. 2012). Thus, we hypothesize that PGE_2_ effects are self-limiting because of desensitization caused by prolonged agonist activation of EP receptors. Thus, identifying the EP receptors involved in pulmonary fibroblast cAMP signaling, establishing whether they desensitize upon prolonged agonist exposure, and identifying the mechanism involved in any desensitization are all critical for developing novel therapeutic approaches for PF.

GPCRs that signal via cAMP are desensitized by two primary mechanisms: GRK/β-arrestin–mediated uncoupling, internalization, and eventual downregulation of receptors, and/or increased expression of PDE activity. EP_2_ receptors have a short C-terminal tail, lack the residues for GRK phosphorylation, and thus do not internalize (Desai et al. 2000). Moreover, later studies established that EP_2_ receptors are not subject to other classical mechanisms of GRK/arrestin-mediated desensitization of GPCRs (Penn et al. 2001; Deshpande et al. 2008). Conversely, EP_4_ receptors possess classical regulatory sites on their C-termini and so undergo β-arrestin–mediated internalization (Desai and Ashby 2001). One or both of these EP receptors may be expressed in human fetal lung (HFL)-1 cells, so diminished PGE_2_-stimulated cAMP levels could result from either decreased receptor activation via receptor desensitization or increased PDE activity or both. In the current study, we tested whether long-term PGE_2_ exposure causes desensitization, identified the specific EP receptor subtypes inducing this desensitization, and established whether upregulation of specific PDE isoforms is responsible for the desensitization.

## Materials and methods

### Cell culture

HFL-1 fibroblasts (ATCC, Manassas, VA, USA) were grown in Ham’s F12K medium (Thermo Fisher Scientific, Waltham, MA, USA) with 10% fetal bovine serum (Atlanta Biologicals, Flowery Branch, GA, USA) and 1% antibiotic-antimycotic solution. Cells with a passage number of 13 to 25 were used and were grown to 70–90% confluency for all studies. Cells were grown in an incubator at 37 °C and 5% CO_2_ levels. Medium was aspirated and cells were washed once with PBS. After aspiration of PBS, 5 mL of 0.25% trypsin-EDTA (Thermo Fisher Scientific, Waltham, MA, USA) was added for 5 min to induce cell detachment. Media was added to the flask and gently mixed to suspend detached cells, transferred to tubes, centrifuged at 500g for 5 min, then washed to remove trypsin. The cell pellet was resuspended in growth medium, and approximately 9000 sells were plated per well on 96-well plates. Cells were pretreated with PGE_2_, other drugs, or vehicle prior to experiments by adding 10× concentration of drug to growth media. Medium was aspirated after 24 h, and each well was washed once with warm Dulbecco’s phosphate-buffered saline before performing cADDis assay for cAMP.

### cADDis assay for cAMP

The cADDis assay was purchased from Montana Molecular (Bozeman, MT, USA). HFL-1 cells were incubated with a recombinant mammalianized baculovirus (BacMam) engineered to express a genetically modified protein that is a fusion of EPAC, a cAMP effector, with the green fluorescent protein (GFP). This protein serves as a convenient and quantitative cAMP sensor. In its unbound form, the protein fluoresces, but its fluorescence is quenched upon binding of cAMP. When seeding the cells in a 96-well plate, the following components were added: 138 μL of cells plus media, 40 μL BacMam solution, and 2 μL of trichostatin A (an inhibitor of histone deacetylase) (Sigma®, St. Louis, MO, USA). In the 96-well plate format, approximately 8900 cells were seeded per well and 2.95 × 10^8^ viral particles per well were added. Trichostatin A stock concentration was 100 μM, giving a final concentration of 1 μM in the final volume of 200 μL per well. Transduced HFL-1 cells were incubated for 24 h at 37 °C and 5% CO_2_ levels. After incubation, medium was aspirated and replaced with 180 μL per well of 1× Dulbecco’s phosphate-buffered saline without calcium and magnesium (Thermo Fisher Scientific, Waltham, MA, USA). The 96-well plate was then covered with aluminum foil and incubated at 37 °C for 30 min. The plate was placed in a SpectraMax M5 plate reader (Molecular Devices, San Jose, CA, USA), and fluorescence was read from the bottom of the plate with an excitation wavelength of 494 nm and an emission wavelength of 522 nm for 5 min. Once variability of fluorescence was ≤ 5% in all wells, different concentrations ofPGE_2_ or other agents were added with a multi-pipette and the changes in fluorescence were measured every 30 s for 30 min.

Data from kinetic studies of fluorescence were normalized to the original level of fluorescence (Δ*F*/*F*_0_) and then fit to a single-site decay model using GraphPad Prism 7.0 (GraphPad Software Inc., San Diego, CA, USA). The plateau of each decay curve was then used as the maximal response to that given concentration of drug (*E*_max_), and concentration-response curves were generated using nonlinear regression analysis (log [agonist] vs. response, variable slope, four parameters) using GraphPad Prism 7.0. Concentration-response curves in multiple conditions were compared using two-way analysis of variance (2-way ANOVA) with Tukey’s multiple comparison test, and significance (*p* value) is reported for the effect of different conditions. Statistical comparison tests (*t* tests, 1-way and 2-way ANOVA) were performed using GraphPad Prism.

### Assay of basal cAMP

HFL-1 cells were washed three times with serum- and NaHCO_3_-free DMEM supplemented with 20 mM HEPES, pH 7.4 (DMEH), then incubated for 30 min at 37 °C. Medium was aspirated, and 250 μL of trichloroacetic acid (TCA, 7.5% *w*/*v*) was added to each well. cAMP content in TCA extracts was determined by EIA (Cayman Chemical) following the manufacturer’s instructions. The amount of cAMP was normalized to the amount of protein per sample as determined using a dye-binding protein assay (Bio-Rad).

### Real-time quantitative reverse transcription polymerase chain reaction

After 48-h incubation with the lentivirus, medium was aspirated, cells were washed once in 1× PBS, and buffer RLT was added to disrupt cell membranes. Cells were collected and RNA was isolated using RNeasy columns with a QIAcube robot (Qiagen). RNA was quantified using the NanoDrop 2000/2000c (Thermo Fisher). Reverse transcription was performed on 400 ng of RNA samples. All steps followed the SuperScript® III First-Strand Synthesis System for RT-PCR protocol (Thermo Fisher). The steps included a denaturation step at 65 °C for 5 min in the RNA/primer mixture, containing 50 ng/μL of random hexamers. Complementary DNA (cDNA) synthesis mixture was added to the RNA/primer mixture and incubated for 10 min at 25 °C, followed by 50 min at 50 °C. The reaction was terminated at 85 °C for 5 min, then chilled on ice. Finally, 1 μL of RNase H was added to each tube followed by incubation for 20 min at 37 °C. cDNA samples were either used immediately for quantitative reverse transcription polymerase chain reaction (qRT-PCR) experiments or stored at − 20 °C.

TaqMan® Gene Expression Assays (Thermo Fisher) were used for qRT-PCR reactions. The ratio of components for each reaction was as follows: 1 μL 20× TaqMan® Gene Expression Assay, 10 μL 2× TaqMan® Gene Expression Mater Mix, 4 μL cDNA (1 ng to 100 ng), and 5 μL of RNase-free water. The thermal cycler settings were as follows: step 1, 50 °C for 2 min; step 2, 95 °C for 10 min; step 3, 95 °C for 15 s; and step 4, 60 °C for 1 min. Steps 3 and 4 were repeated for a total of 40 cycles. The housekeeping genes GAPDH, β-actin, and CDKN1A were used to normalize for differences in RNA samples. PCR data were analyzed using the 2^[−ΔΔCt]^ method, where Ct is the cycle threshold.

### PDE activity assays

PDE assays were performed according to the methods of Bauer and Schwabe (1980). Following pretreatments, cells were washed, scraped from the dishes, and lysed by sonication. Cell lysates were pre-incubated for 5 min with inhibitors or vehicle. Assay mix containing [^3^H] cAMP was added followed by incubation for 25 min at 37 °C with shaking. The reaction was stopped by adding 0.2 N HCl, and samples were placed on ice for at least 10 min. *Crotalus atrox* snake venom–derived 5′-nucleotidase was added, and samples were incubated for 20 min at 37 °C. The assay mixture was then added to QAE-Sephadex A25 columns, and nucleosides were eluted with 30 mM ammonium formate. [^3^H] adenosine formation was quantified by liquid scintillation counting and taken as total PDE activity. In some experiments, roflumilast (1 μM) or 3-isobutyl-1-methylxanthine (IBMX) (1 mM) was included during the PDE assays to assess PDE4 and total PDE activities, respectively.

### Statistics

Statistical comparison tests (one-way and two-way analyses of variance) were performed with GraphPad Prism 7.0. Tukey’s multiple comparison test was performed where appropriate.

## Results

**Fig. 1:**
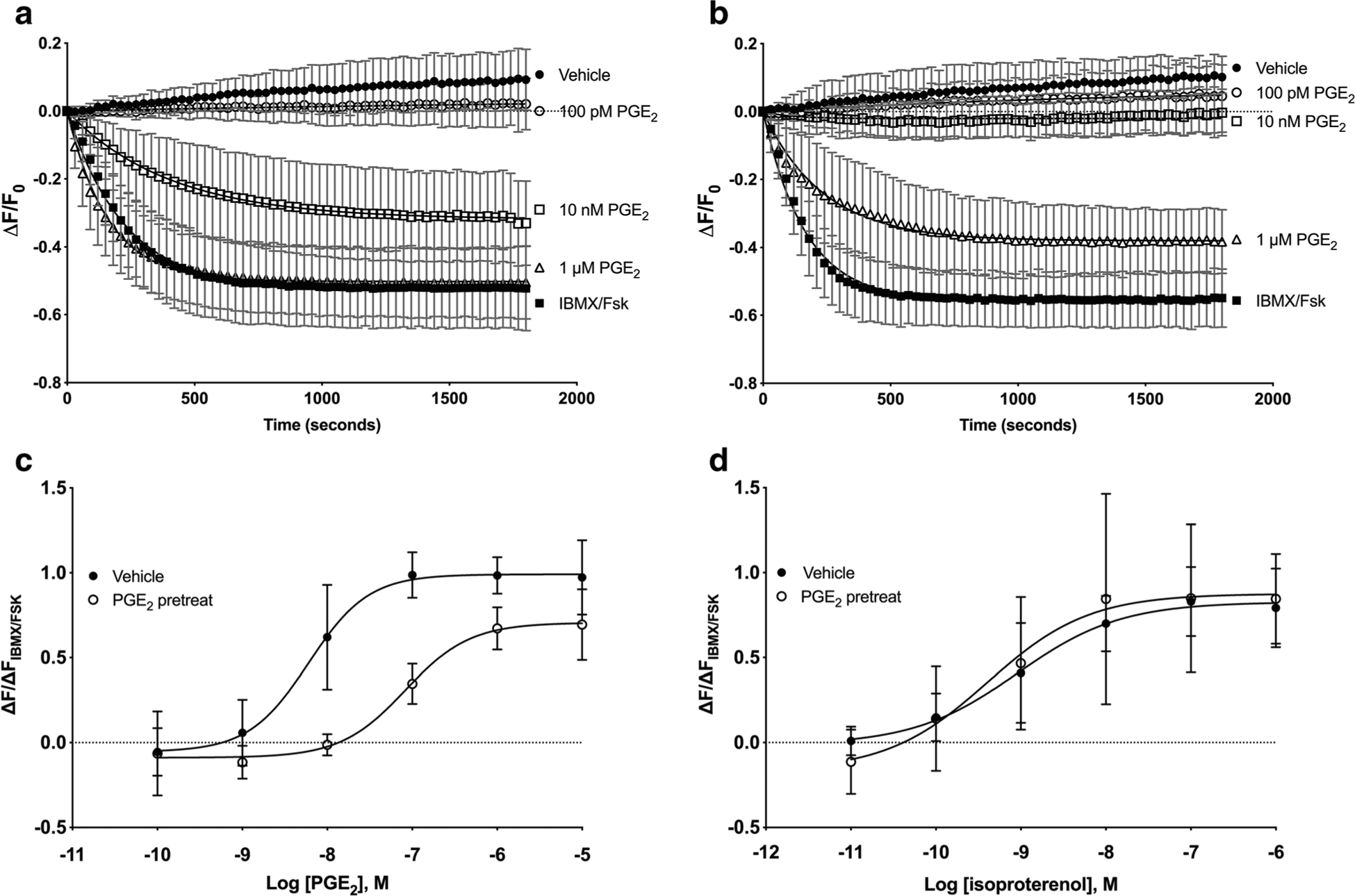
Twenty-four-hour pretreatment with PGE_2_ induces desensitization of cAMP responses to PGE_2_ but not isoproterenol. HFL-1 cells were treated with vehicle or 100 nM PGE_2_ for 24 h and washed, then acute responses to PGE_2_ were measured using the cADDis cAMP sensor. Fluorescent decay curves are shown for three concentrations of PGE_2_ in vehicle-pretreated (**a**) or PGE_2_-pretreated (**b**) cells. The plateau from each decay curve was normalized to the maximal response to 200 mM IBMX plus 1 μM forskolin and plotted as a concentration-response curve. Concentration-response curves to PGE_2_ (**c**) or isoproterenol (**d**) are shown in both vehicle and 100 nM PGE_2_-pretreated cells. Data are mean ± SEM of *n* = 3–10. **c** Significant (*p* < 0.0001) and **d** not significant (*p* = 0.070) by 2-way ANOVA

To determine whether prolonged exposure of HFL-1 cells to PGE_2_ causes desensitization of subsequent PGE_2_ responses, cells were pretreated with 1 nM, 10 nM, or 100 nM PGE_2_ for 24 h. Cells were then washed to remove the pretreatment drug, and cAMP responses to various concentrations of PGE_2_ were measured using the cADDis assay. The change of fluorescence caused by each concentration ofPGE_2_ was measured for 30 min, and the data was fit to a one-site decay model (Fig. 1a, b). The plateau of each response was then normalized to the maximal response elicited by forskolin plus IBMX and plotted to generate a concentration-response curve to PGE_2_ for each pretreatment condition. PGE_2_ stimulated cAMP production in vehicle-treated cells with a log EC_50_ value of −7.40 ±0.16 (Fig. 1c, Table 1). By contrast, PGE_2_ responses in cells pretreated with 1 nM PGE_2_ required moderately higher concentrations (log EC_50_ = − 7.10 ± 0.25) and cells pretreated with 10 nM PGE_2_ displayed PGE_2_ responses that required even higher concentrations (log EC_50_ = − 6.79 ± 0.20, Table 1). Pretreatment with 100 nM PGE_2_ induced the largest rightward shift of the PGE_2_ concentration-response curve to − 6.09 ± 0.15 (Fig. 1c, Table 1). None of the pretreatment conditions produced a significant reduction in *E*_max_. Therefore, pre-exposure to PGE_2_ causes reductions in the potency of PGE_2_ for increasing cAMP levels.

We assessed basal cAMP levels in cells pretreated with either vehicle or 100 nM PGE_2_ for 24 h. Because biosensors such as cADDis can only display changes in cAMP levels, we lysed cells after treatment and measured cAMP levels using an EIA. Basal cAMP level in vehicle-treated cells was 57.7 ±15.4 fmol/mg protein while basal cAMP level in PGE_2_-pretreated cells was 57.9 ± 13.8 fmol/mg protein (not significant *p* = 0.815 by Student’s *t* test, data not shown). Thus, PGE_2_ pretreatment does not alter basal levels of cAMP in the bulk cytosol.

We investigated whether a 24-h pretreatment with PGE_2_ alters responses mediated by other receptors. cAMP levels stimulated by the β-adrenoceptor (β-AR) agonist isoproterenol were measured in cells pretreated with 100 nM PGE_2_ or vehicle for 24 h. In contrast to the 22-fold rightward shift in the PGE_2_ concentration-response curve in PGE_2_-pretreated cells, responses mediated by isoproterenol were not right-shifted by PGE_2_ pretreatment, with log EC_50_ values of − 9.05 ± 0.33 and − 9.39 ± 0.60 for vehicle and PGE_2_ pretreatment, respectively (Fig. 1d). Thus, PGE_2_ pre-exposure does not cause desensitization of β-AR–mediated cAMP signaling.

**Fig. 2:**
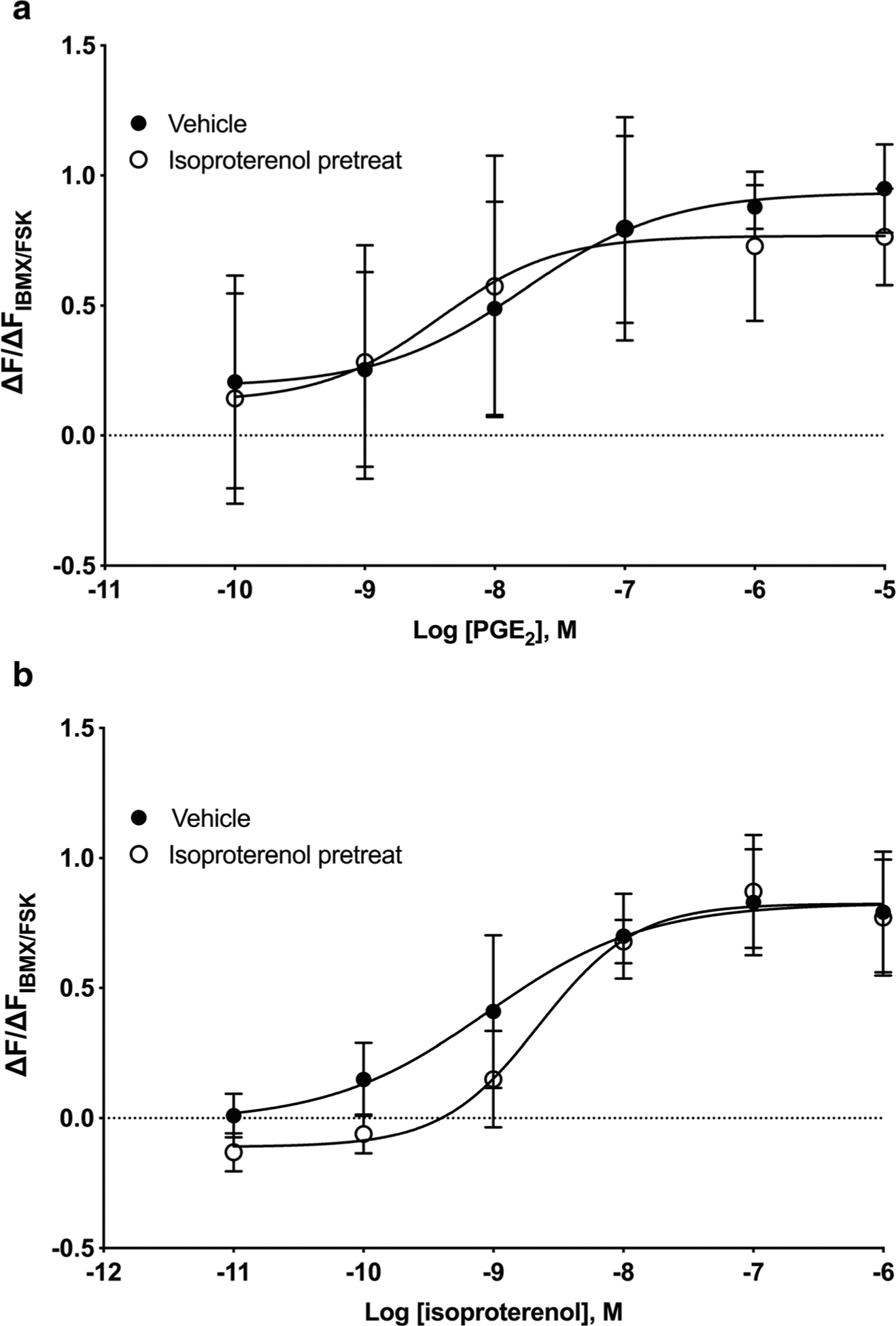
Twenty-four-hour pretreatment with isoproterenol does not induce desensitization of PGE_2_ cAMP responses. HFL-1 cells were treated with 100 nM isoproterenol for 24 h then washed, and acute responses to PGE_2_ (**a**) or isoproterenol (**b**) were measured. cAMP levels were measured as a function of change in fluorescence normalized to the change in fluorescence of maximal response stimulated by 1 μM forskolin plus 200 μM IBMX using the cADDis assay. Data are mean ± SEM of *n* = 5. **a** Not significant (*p* = 0.612) and **b** significant (*p* = 0.0318) by 2-way ANOVA

**Fig. 3:**
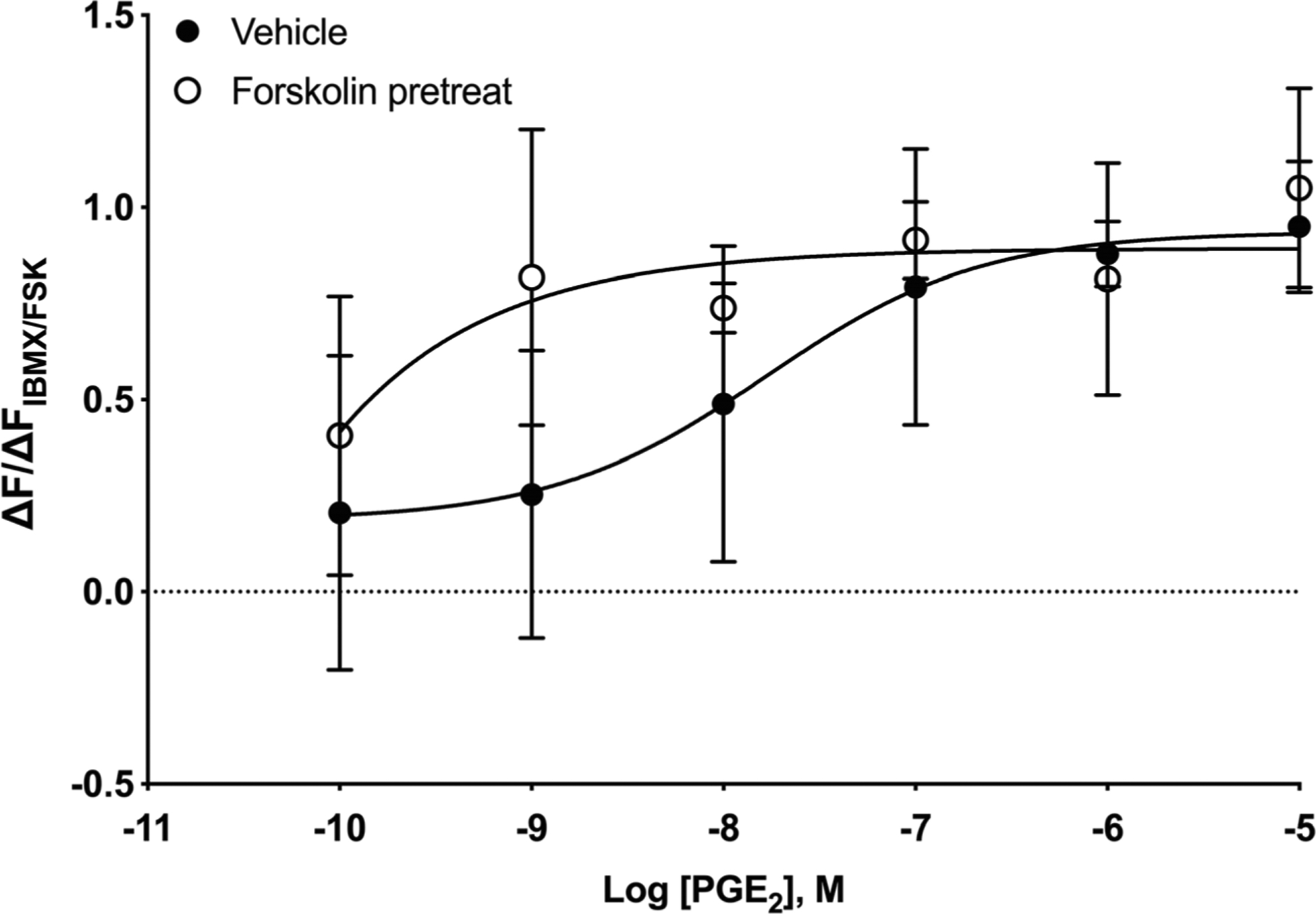
Twenty-four-hour pretreatment with forskolin does not induce desensitization of PGE_2_ cAMP responses. HFL-1 cells were treated with 1 μM forskolin for 24 h then washed, and acute responses to PGE_2_ were measured. cAMP levels were measured as a function of change in fluorescence normalized to the change in fluorescence of maximal response stimulated by 1 μM forskolin plus 200 μM IBMX using the cADDis assay. Data are mean ± SEM of *n* = 5. Not significant (*p* = 0.287) by 2-way ANOVA

To establish if decreased responsiveness to PGE_2_ occurs upon pretreatment with other cAMP-elevating agents, the effects of pretreating cells with either vehicle, 100 nM isoproterenol, or 1 μM forskolin (a direct activator of AC) were assessed. Log EC_50_ values for PGE_2_ stimulation were − 7.78 ± 0.52 and − 8.44 ± 0.69 for vehicle and isoproterenol pretreatment, respectively (Fig. 2a). cAMP responses to isoproterenol displayed log EC_50_ values of − 9.05 ± 0.33 and −8.65 ± 0.15 for vehicle and isoproterenol, respectively (Fig. 2b). Thus, pretreatment with isoproterenol induces desensitization of β-AR responses but does not decrease responses to PGE_2_. Pretreatment of HFL-1 cells with 1 μM forskolin for 24 h also did not cause desensitization of PGE_2_ responses, with log EC_50_ values of − 7.78 ± 0.52 and − 9.36 ±3.54 for vehicle and forskolin pretreatment, respectively (Fig. 3). The small increase in PGE_2_ potency observed in these studies may be due to residual forskolin from the pretreatment phase that could not be washed out.

**Fig. 4:**
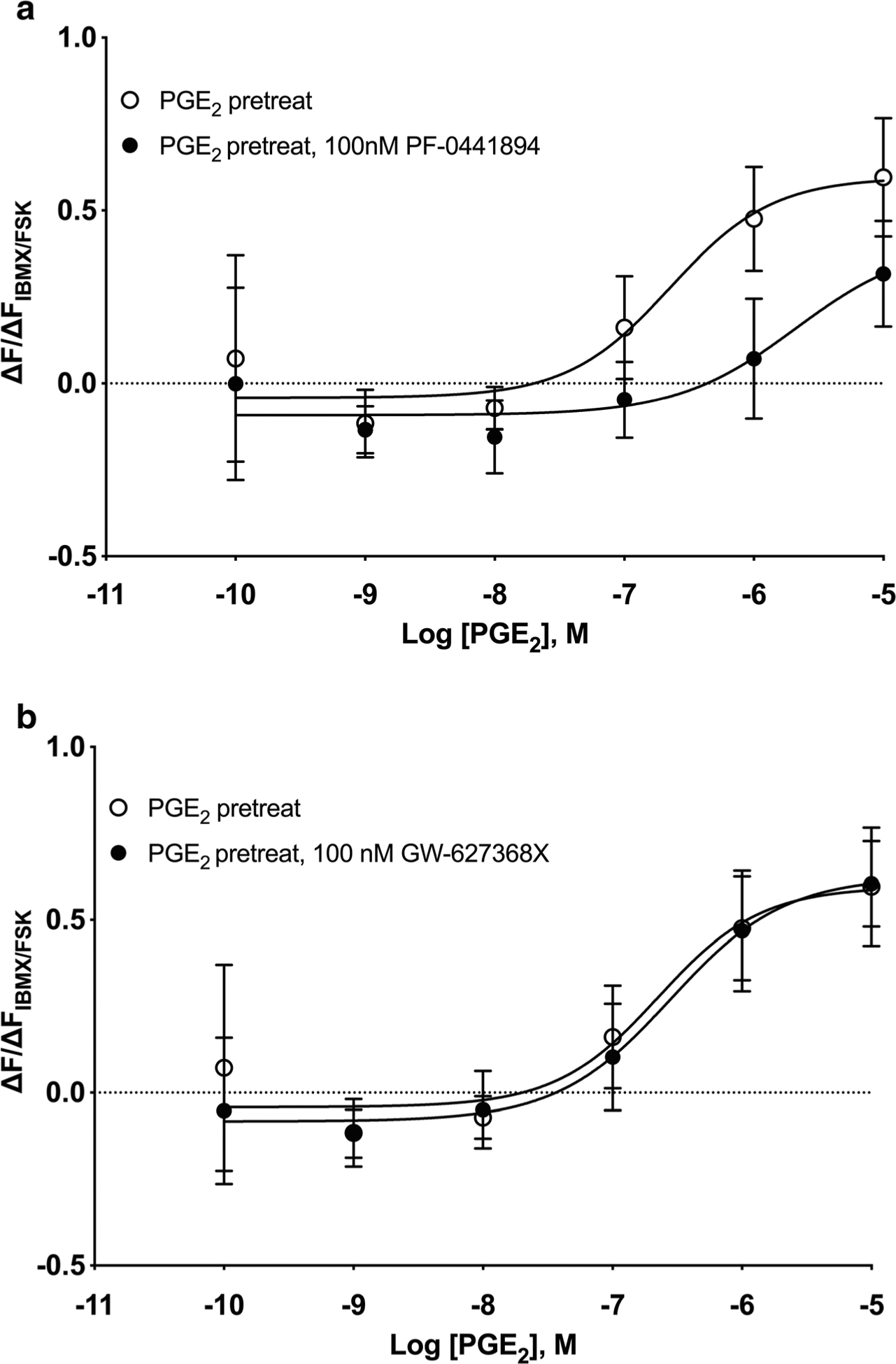
PGE_2_-stimulated cAMP responses in HFL-1 cells are mediated by EP_2_ not EP_4_ receptors. HFL-1 cells were pretreated with 100 nM PGE_2_ for 24 h. **a** 10 min prior to measuring cAMP responses to PGE_2_ with addition of either vehicle or 100 nM PF-0441894 (EP_2_-selective antagonist). **b** 10 min prior to measuring cAMP responses to PGE_2_ with addition of either vehicle or 100 nM GW-627368X (EP_4_-selective antagonist). cAMP levels were measured as a function of change in fluorescence normalized to the change in fluorescence of maximal response stimulated by 1 μM forskolin plus 200 μM IBMX using the cADDis assay. Data are mean ± SEM of *n* = 6. **a** Significant (*p* < 0.0001) and **b** not significant (*p* = 0.421) by 2-way ANOVA

PGE_2_ can increase cAMP by activation of EP_2_ or EP_4_ receptors. To assess which of these two EP receptor types mediates PGE_2_-stimulated cAMP responses, we examined the effect of PF-0441894 (EP_2_ receptor–specific antagonist) or GW-627368X (EP_4_ receptor–specific antagonist) on PGE_2_-stimulated cAMP responses. PF-0441894 (100 nM) shifted the PGE_2_ concentration-response curve by nearly 10-fold the right, while GW-627368X (100 nM) did not cause a rightward shift (Table 2). We also examined the effects of both PF-0441894 and GW-627368X on PGE_2_-stimulated cAMP responses in cells that had been pretreated with PGE_2_ for 24 After a 24-h pretreatment with either PGE_2_ or vehicle, cells were washed and equilibrated for 30 min, and then each antagonist was added. Ten minutes after addition of antagonist, various concentrations of PGE_2_ were added and cAMP responses were measured for 30 min. Unlike our results Figs. 1, 2, and 3, PGE_2_ pretreatment caused a reduction PGE_2_ maximal response in addition to a rightward shift (vehicle 1.06 ± 0.10, PGE_2_-pretreated 0.60 ± 0.09). Just as vehicle-pretreated cells, PF-0441894 shifted PGE concentration-response curves to the right (Fig. 4a, Table 2 but GW-627368X did not (Fig. 4b, Table 2). Based on published K_i_ values, 100 nM PF-0441894 occupies 95% of EP receptors while 100 nM GW-627368X occupies just 1.2% EP_2_ receptors (Wilson et al. 2006; af Forselles et al. 2011 These results suggest that EP_2_ receptors, but not EP_4_ receptors, mediate the PGE_2_-induced increase in cAMP and that pretreatment with PGE_2_ for 24 h does not alter this receptor response profile.

**Fig. 5:**
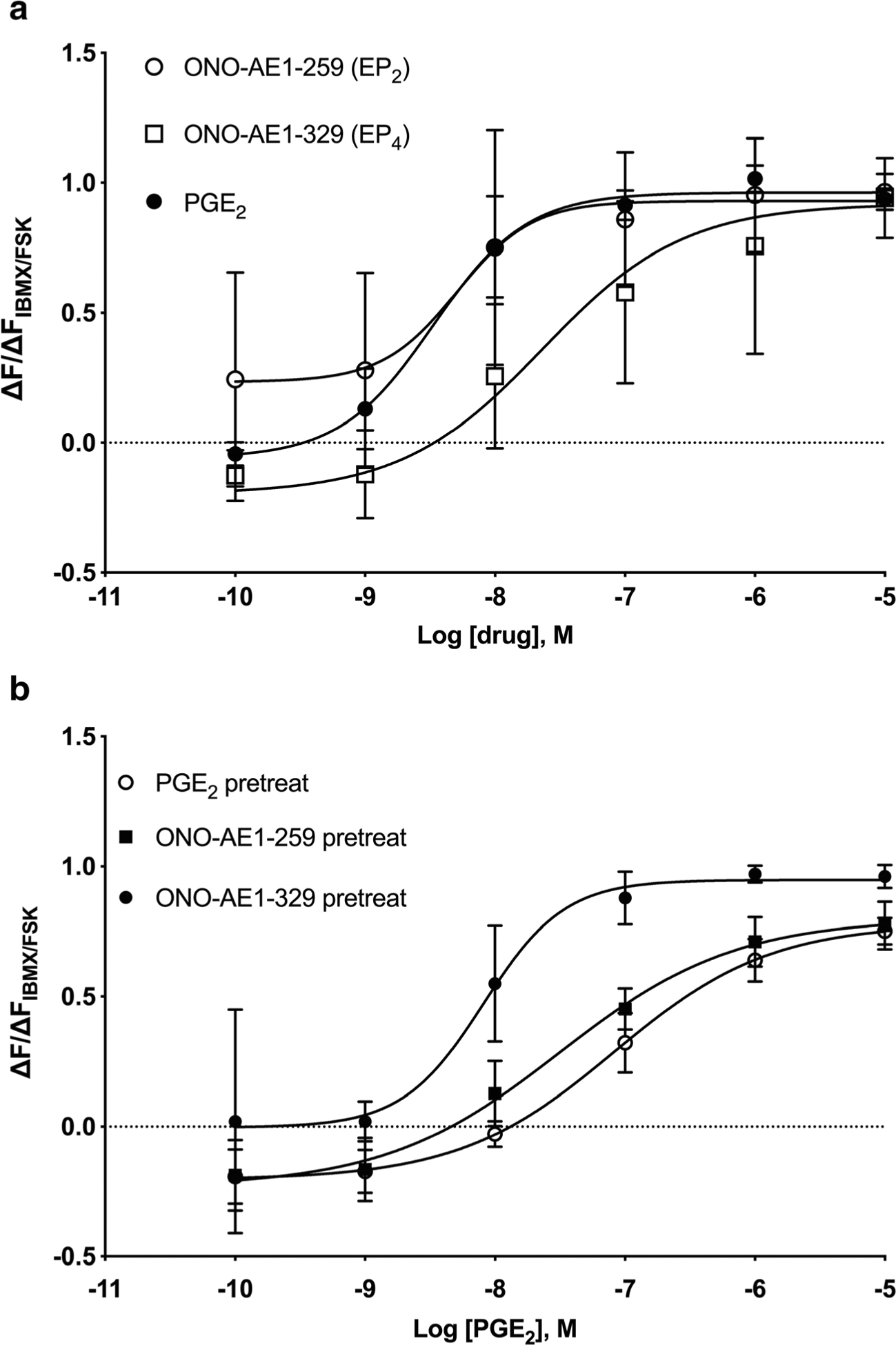
PGE_2_ responses are desensitized following the 24-h pretreatment with an EP_2_R-selective agonist but not by an EP_4_R-selective agonist. **a** Acute cAMP responses in HFL-1 cells to various concentrations of PGE_2_, ONO-AE1–259 (EP_2_ agonist), or ONO-AE1–329 were measured. **b** HFL-1 cells were treated with 100 nM PGE_2_, 100 nM ONO AE1–259 (EP_2_ agonist), or 100 nM ONO-AE1–329 (EP_4_ agonist) for 24 h then washed, and acute responses to PGE_2_ were measured. cAMP levels were measured as a function of change in fluorescence normalized to the change in fluorescence of maximal response stimulated by 1 μM forskolin plus 200 μM IBMX using the cADDis assay. Data are mean ± SEM of *n* = 4. ONO-AE1–259 is not significant (*p* = 0.264), while ONO-AE1–329 is significant (*p* < 0.0001) from PGE_2_ by 2-way ANOVA

To understand which EP receptors mediate PGE_2_-induced desensitization, the receptor subtype–selective agonists ONO-AE1–259 (EP_2_) and ONO-329 (EP_4_) were tested for their abilities to induce desensitization. Both ONO-AE1–259 and ONO-AE1–329 increased cAMP levels acutely in HFL-1 cells, but ONO-AE1–259 did so with a log EC_50_ value of −8.29 (similar to the potency of PGE_2_, log EC_50_ value of −8.48) while ONO-AE1–329 was far less potent (log EC_50_ value of − 7.49, Fig. 5a). Cells were pretreated with vehicle, 100 nM PGE_2_, 100 nM ONO-AE1–259, or 100 nM ONO-AE1–329 for 24 h and washed, and then cAMP responses to PGE_2_ were measured. ONO-AE1–259 at 100 nM is predicted to occupy 97% of EP_2_ receptors but just 4.1% of EP_4_ receptors based on published *K*_i_ values (Suzawa et al. 2000; Ganesh 2014). ONO-AE1–329 at 100 nM is predicted to occupy 4.7% of EP_2_ receptors but 91% of EP_4_ receptors (Suzawa et al. 2000). ONO-AE1–259 pretreatment shifted PGE_2_ responses rightward by 4.0 ± 1.2-fold (*n* = 4) compared to vehicle pretreatment, with log EC_50_ values of − 7.51 ± 0.18 and − 8.00 ± 0.09, respectively (Fig. 5b). This degree of desensitization was nearly equivalent to the 8.0 ± 2.2-fold shift induced by pretreatment with 100 nM PGE_2_ in the same experiments. ONO-AE1–329 pretreatment did not induce a change compared to vehicle pretreatment, with PGE_2_ log EC_50_ values of − 8.09 ± 0.17 and − 8.00 ± 0.09, respectively. Together, these data indicate that EP_2_ receptors are responsible for desensitization of PGE_2_ responses in HFL-1 cells. Our findings are supported by other studies that report the relative abundance of mRNA levels among G_s_-coupled GPCRs in HFL-1 cells. Using qRT-PCR and cAMP measures with PDE inhibitors, Roberts et al. (2018) determined the relative abundance of receptors to be IP receptors > EP_2_ receptors >>> EP_4_ receptors. RNA-Seq studies of HFL-1 cells reported in the Gene Expression Omnibus show the relative abundance of mRNA to be EP_2_ receptors > IP receptors = EP_4_ receptors (GEO accession GSE73555). Thus, while the expression level of IP receptors is debatable, it is clear that EP_2_ receptors are expressed at much higher levels than EP_4_ receptors in HFL-1 cells.

**Fig. 6:**
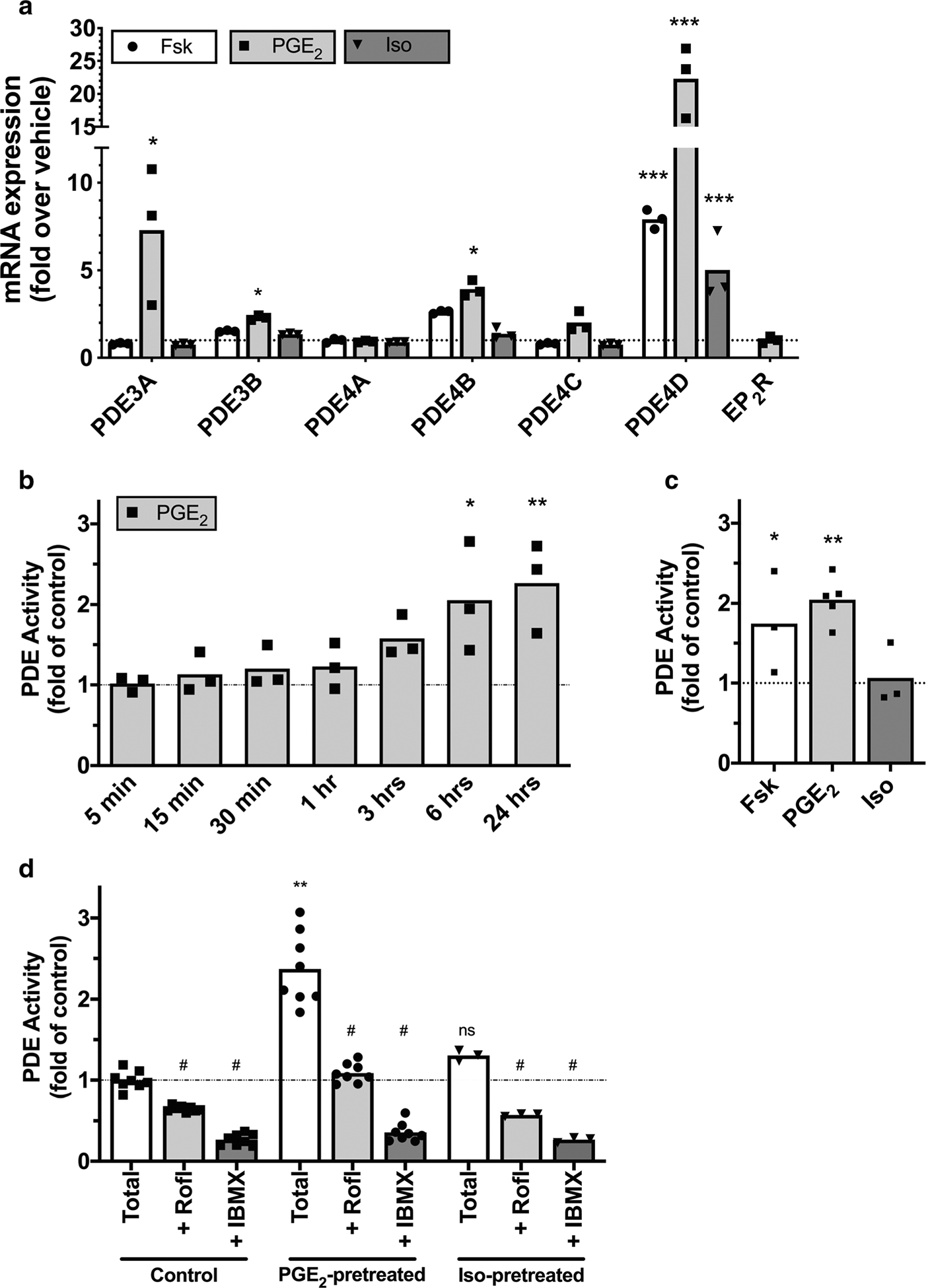
PDE isoform expression and activity is increased by PGE_2_ but not isoproterenol pretreatment. **a** HFL-1 cells were incubated with vehicle, 1 μM forskolin, 100 nM PGE_2_, or 100 nM isoproterenol for 24 h, then the expression of PDE isoform mRNAwas assessed by quantitative RT-PCR. Expression of EP_2_ receptors was assessed in the same way following PGE_2_ pretreatment. **b** Cells were incubated for the indicated times with vehicle or 100 nM PGE_2_, then PDE enzyme activity in cell lysates was assayed. **c** Cells were incubated for 24 h with vehicle, 30 μM forskolin, 100 nM PGE_2_, or 1 μM isoproterenol, then PDE enzyme activity in cell lysates was assayed. **d** Cells were incubated for 24 h with vehicle, 100 nM PGE_2_, or 1 μM isoproterenol, then PDE enzyme activity in cell lysates was assayed in the presence of either vehicle (total), roflumilast (1 μM), or IBMX (1 mM). Data are expressed as the fold change over vehicle-treated cells (dotted line). Bars show means from *n* = 3–8 with individual data points plotted in symbols. *p < 0.05, **p < 0.01, and ***p < 0.0001 by two-way ANOVA (**a, d**) or one-way ANOVA (**b, c**) as compared to vehicle. ^#^*p* < 0.05 by two-way ANOVA as compared to total activity in the same condition

cAMP levels are also regulated by phosphodiesterase activity, which hydrolyzes cAMP to AMP (Kohyama et al. 2002b), so increases in PDE activity provide another possible explanation for the desensitization we observed. HFL-1 cells primarily express PDE3B, PDE4A, PDE4B, PDE4C, PDE4D, PDE7A, PDE7B, and PDE8A (GEO accession GSE73555) (Kohyama et al. 2002b). qRT-PCR experiments were performed to determine whether the expression of any of these PDE isoforms is altered by PGE_2_ pretreatment. Cells were pretreated with 1 μM forskolin, 100 nM isoproterenol, or 100 nM PGE_2_ for 24 h, and changes in PDE subtype expression were quantified by qRT-PCR. Significant upregulation of PDE3A, PDE3B, PDE4B, and PDE4D occurred following pretreatment with each of these cAMP-elevating agents (Fig. 6). Of these isoforms, PDE3A was selectively upregulated by PGE_2_ pretreatment (7.3-fold), with forskolin and isoproterenol having no effect on PDE3A. PGE_2_ and forskolin pretreatment upregulated PDE4B expression (2.6- and 3.9-fold, respectively), but isoproterenol had no effect (1.3-fold). PDE4D was upregulated significantly by all three drugs, but more by PGE_2_ (22.3-fold) than forskolin or isoproterenol (7.9- and 5.0-fold, respectively). While a previous report suggests that a 12-h pretreatment with 1 μM PGE_2_ can downregulate expression of both EP_2_R and EP_4_R when exogenously expressed in Chinese hamster ovary cells (Nishigaki et al. 1996), we did not observe any change in EP_2_R mRNA levels following a 24-h pretreatment with 100 nM PGE_2_ (Fig. 6a).

PDE activity assays performed in cell lysates from HFL-1 cells pretreated with 100 nM PGE_2_ confirm that the observed increase in PDE mRNA translates to increased cAMP hydrolyzing activity. We treated cells for various time points from 5 min to 24 h and found significant increases in bulk PDE activity after 6 h or more of PGE_2_ treatment as compared to vehicle (Fig. 6d). We then treated cells with either vehicle, 30 μM forskolin, 100 nM PGE_2_ or 1 μM isoproterenol for 24 h and examined bulk PDE activity. Forskolin and PGE_2_, but not isoproterenol, significantly increased PDE activity (Fig. 6c). Inclusion of the PDE4 family inhibitor roflumilast (1 μM) or the broad-spectrum PDE inhibitor IBMX (1 mM) significantly reduced PDE activity in vehicle (control) and in PGE_2_- and isoproterenol-pretreated cells (Fig. 6d). Therefore, PGE_2_ pretreatment significantly upregulates PDE activity while isoproterenol does not. Much of the upregulated PDE activity appears sensitive to a PDE4 inhibitor.

**Fig. 7:**
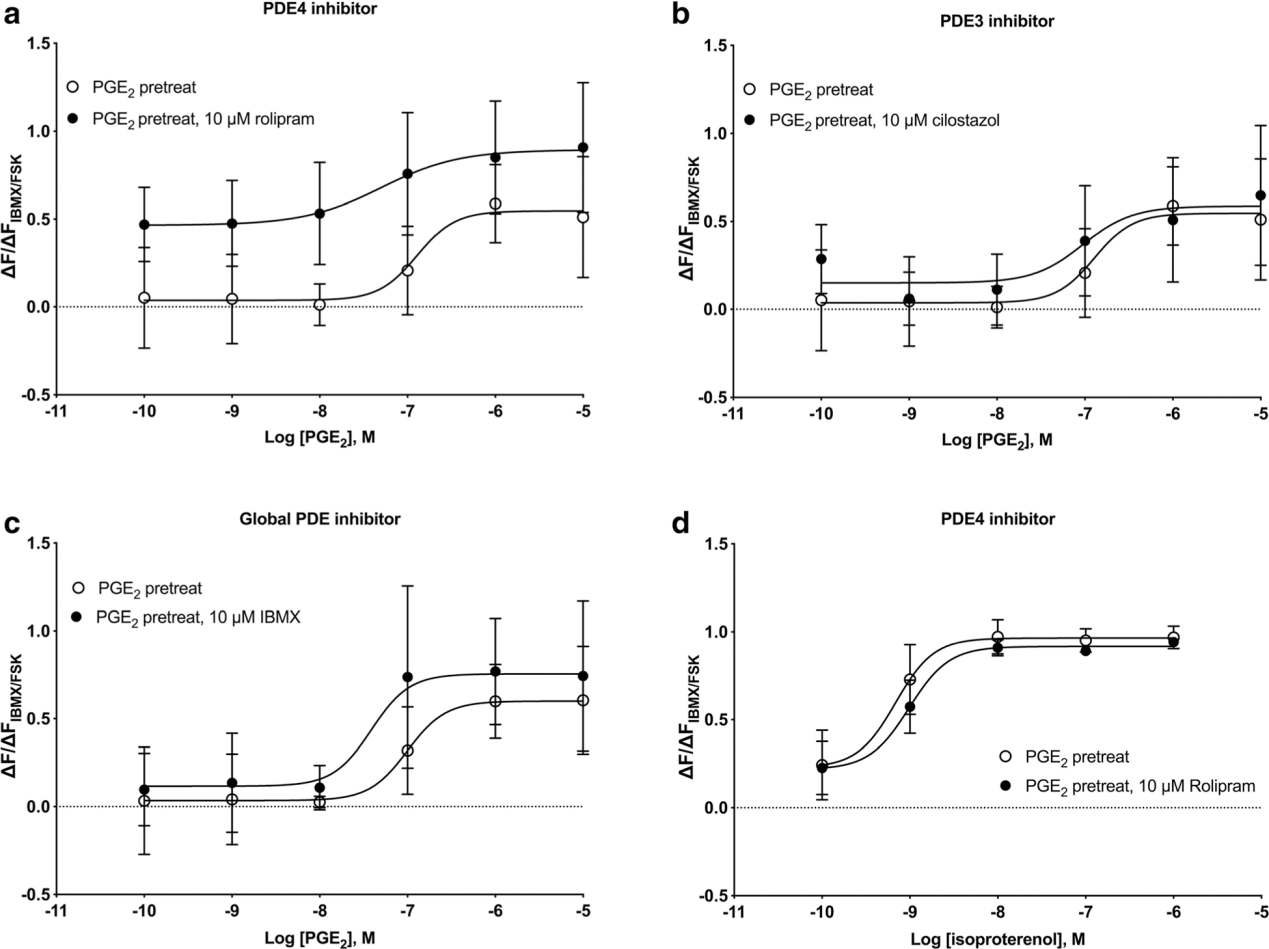
Inhibition of PDE4, but not PDE3, re-sensitizes PGE_2_-mediated cAMP responses. HFL-1 cells were pretreated with 100 nM PGE_2_ for 24 h, prior to measuring cAMP responses to either PGE_2_ (**a**–c) or isoproterenol (**d**). Either vehicle, 10 μM rolipram (PDE4 inhibitor, panels **a** and **d**), 10 μM cilostazol (PDE3 inhibitor, panel **b**), or 10 μM IBMX (broad PDE isoform inhibitor, panel **c**) was added 10 min prior to addition of PGE_2_ or isoproterenol. cAMP production was measured as a function of change in fluorescence normalized to the change in fluorescence of maximal response stimulated by 1 μM forskolin and 200 μM IBMX using the cADDis assay. Data are mean ± SEM of *n* = 3. **a** Significant (*p* < 0.0001), **b** not significant (*p* = 0.286), **c** not significant (*p* = 0.114), and **d** not significant (*p* = 0.259) by 2-way ANOVA

We used PDE3- and PDE4-specific inhibitors to examine if these isozymes are involved in mediating desensitization of PGE_2_ cAMP responses. HFL-1 cells were pretreated for 24 h with 100 nM PGE_2_, washed, and then incubated with 10 μM rolipram (a PDE4 family inhibitor), 10 μM cilostazol (a PDE3 family inhibitor), or 10 μM IBMX for 10 min prior to measuring cAMP levels in response to various concentrations of PGE_2_. The PDE_4_-selective inhibitor rolipram was able to reverse the desensitization caused by PGE_2_ pretreatment by shifting the concentration-response curve leftward by 2.8-fold (Fig. 7a). The PGE_2_ log EC_50_ value in the PGE_2_-pretreated condition was − 6.85 ± 0.25 while the log EC_50_ value in the presence of 10 μM rolipram was − 7.30 ± 0.11. The addition of the PDE3-selective inhibitor cilostazol at 10 μM had no effect, with the PGE_2_ log EC_50_ value of − 7.03 ± 0.39 when 10 μM cilostazol was present (Fig. 7b). IBMX, a nonselective inhibitor of all PDE isozymes except PDE8, induced a small leftward shift that was not statistically significant. The PGE_2_

log EC_50_ value in the PGE_2_-pretreated condition was − 7.00 ± 0.22 while the log EC_50_ value in the presence of 10 μM IBMX was − 7.47 ± 0.62 (Fig. 7c). A lower concentration of IBMX was used in these studies since higher concentrations stimulated increases in cAMP that nearly saturated the cADDis biosensor. Rolipram had no effect on isoproterenol responses in cells pretreated with PGE_2_, implying that the increased PDE activity does not regulate β-AR signaling (Fig. 7d). Taken together, these results are consistent with the idea that PGE_2_ pretreatment induces expression of PDE4 isozyme(s) that regulates cAMP signals stimulated by PGE_2_ but not those stimulated by β-AR.

## Discussion

Evidence that PGE_2_ produces antifibrotic effects is relatively clear in the literature (Kolodsick et al. 2003; White et al. 2005; Bozyk and Moore 2011). PGE_2_ is produced as part of the normal injury response, to promote lung homeostasis and to inhibit fibrotic processes and help promote alveolar epithelial cell regeneration and thus restore airway barrier function (Wilborn et al. 1995; Bozyk and Moore 2011). The signaling pathways that PGE_2_ activates remain attractive targets for therapies in PF. For example, PGE_2_ activates EP receptors that are coupled to the G_s_-AC-cAMP pathway that promote an antifibrotic response (Bozyk and Moore 2011). However, some studies demonstrate diminished COX-2 expression in lung fibroblasts cultured from patients with PF, which would lead to reduced PGE_2_ synthesis (Wilborn et al. 1995). Despite this, PGE_2_ levels remain elevated in fibrotic lungs, so the reason for its limited antifibrotic action in PF is not explained. It is possible that PGE_2_ loses its antifibrotic action following long-term exposure due to attenuated cAMP signaling in lung fibroblasts (Michalski et al. 2012). Because PF is characterized by ongoing airway injury and presumably chronic elevation of PGE_2_ (Fastres et al. 2017), the current study examined how EP receptor signaling is affected by prolonged agonist exposure. Our findings demonstrate that a 24-h exposure of lung fibroblasts to PGE_2_ elicits desensitization of EP_2_ receptor responses. Thus, even if PGE_2_ could be restored to normal levels or administered as a therapeutic agent, this desensitization of EP_2_ receptors would likely inhibit its antifibrotic effects.

The data here show that PGE_2_-induced desensitization of PGE-stimulated cAMP accumulation occurs primarily through activation of EP_2_ receptors and not EP_4_ receptors. Studies with both PGE_2_ receptor subtype–selective antagonists and agonists demonstrated that desensitization of PGE_2_-stimulated cAMP levels requires activation of EP_2_ receptors and provided no evidence for contributions of EP_4_ receptors. Whether EP_4_ receptors are not expressed or rather are not involved in the desensitization response is not clear from our studies. Transcripts for EP_4_ receptors are low but detectible by qRT-PCR and RNA sequencing. PGE_2_ itself also activates EP_3_ and EP_4_ receptors, which can have unwanted profibrotic effects (Bozyk and Moore 2011). More importantly, defining the EP receptor subtype responsible for desensitization allows the future exploration of the molecular signaling components co-localized with EP_2_ receptors in the subcellular compartment in which it resides. This includes PDE isoforms that might be upregulated by prolonged PGE_2_ exposure. Identification of these downstream components co-localized with EP_2_ receptors would increase understanding of how these receptors signal and perhaps provide additional therapeutic approaches that would overcome the loss of signaling caused by chronic agonist exposure.

The mechanism by which EP_2_ receptors desensitize in lung fibroblasts also has not been studied directly. Since EP_2_ receptors do not internalize due to their shorter C-terminus (Desai et al. 2000), we hypothesized that chronic exposure to PGE_2_ might drive an increase in expression of one or more of the cAMP-hydrolyzing PDE isoforms. Data here show that PGE_2_ pretreatment upregulates the expression of PDE3A, PDE3B, PDE4B, and PDE4D mRNA. The changes in expression of PDE3A and PDE4B were highly specific to PGE_2_ pretreatment, as exposure to the β-AR agonist isoproterenol had no effects on these isoforms. The PDE4 inhibitor rolipram but not the PDE3 inhibitor cilostazol was able to partially reverse desensitization to PGE_2_, suggesting that EP_2_ receptor cAMP signals are regulated by a PDE4 isoform in a way that β-AR signaling is not. This mode of regulation has been described in other cells (Bogard et al. 2012; Agarwal et al. 2017), but further studies are needed to confirm the precise PDE isoforms that are active in lung fibroblasts. Previous studies have also implicated PDE4 isoform upregulation following PGE_2_ treatment. Michalski and co-workers (2012) pretreated primary human lung fibroblasts and found PGE_2_ pretreatment attenuated cAMP and chemotactic responses in a manner consistent with upregulation of PDE4. They also found this response was altered in cells isolated from patients with chronic obstructive pulmonary disease, furthering the therapeutic potential of PDE inhibition. We attempted to knockdown the expression of specific PDE isoforms using small interfering RNA (siRNA) approaches but were unable to confirm protein knockdown due to either failure of the siRNA or limitations of available antibodies to detect PDE proteins (data not shown). Therefore, more work is needed to reveal which specific PDE isoforms are responsible for the desensitization to PGE_2_ that we observed.

Another important remaining question is how PGE_2_ pretreatment leads to the increased expression of a specific PDE isoform. Pretreatment with the β-AR agonist isoproterenol had no effect on subsequent PGE_2_ stimulation of cAMP, implying that this effect is limited to PGE and perhaps a subset of other cAMP-elevating agents. Pretreatment with forskolin, which stimulates AC directly to increase cAMP levels throughout the cell, also induced little desensitization. Importantly, isoproterenol and forskolin also induced upregulation of fewer PDE genes, providing a possible explanation for the greater decrease in cAMP accumulation with PGE_2_ pretreatment than for pretreatment with other cAMP-elevating agents. These results imply that either cAMP signaling alone is insufficient for inducing desensitization of EP receptor–stimulated cAMP accumulation or that cAMP signals are highly compartmentalized. cAMP signaling in a very specific subcellular compartment may be required for the desensitization mechanism to be activated, and/or EP_2_ receptors may localize to different compartments than β-AR. In fact, EP_2_ receptors exist in discrete membrane microdomains in various cell types where they can couple to specific AC isoforms, specifically AC2 (Johnstone et al. 2018a). β-AR exists primarily in a different compartment with different AC isozymes and PDE isoforms. Fibroblasts may interpret the localized EP_2_ receptor signal differently and respond by upregulating a specific PDE isoform that selectively regulates cAMP signaling in that domain. This response would leave signaling via β-ARs in their own microdomain unaffected, explaining the results in Figs. 1b and 7d. This hypothesis requires further study but is consistent with that of previous studies and would explain the specific desensitization of PGE_2_ responses observed here. The concept that a single PDE isoform can selectively regulate cAMP signaling in a specific compartment has been previously demonstrated (Johnstone et al. 2018b).

Several limitations to our study should be noted. First, we observed significant variability in the EC_50_ values for PGE_2_ after pretreatment with vehicle or PGE_2_ across different experiments. This results in some studies having large variability in the EC_50_ and/or *E*_max_ values. This may also explain some of the quantitative differences in the PGE_2_ responses seen across different figures. Some of this variability may be due to the difficulty in washing out PGE_2_ (or isoproterenol or forskolin in other experiments) after pretreatment before subsequent responses to PGE_2_ were measured. Our experimental protocol required limited wash steps because more extensive washing negatively affected cell attachment and viability. Residual drug from the pretreatments would be expected to artificially increase potency of subsequent drug additions, as is particularly noted in Fig. 3 when cells were pretreated with forskolin. The variability could also come from differences in expression of the cADDis sensor. Even though we normalize responses to a maximal stimulus, differences in the biosensor levels could alter the observed sensitivity. Nonetheless, the desensitization induced by pretreatment with PGE_2_ was highly reproducible in a qualitative sense and consistently different statistically using 2-way ANOVA. Second, our data do not explain why PGE_2_-induced desensitization was greater than that with pretreatment with the selective EP_2_ receptor agonist ONO-AE1–259 (22-fold vs. 4-fold, respectively), given that our data taken together suggest that the desensitization is mediated selectively by EP_2_ receptor activation. This observation leaves open the possibility that other receptors activated by PGE_2_ but not by ONO-AE1–259 contribute to the induction of desensitization (perhaps EP_1_ and/or EP_3_ receptors coupling to G_q_). The possibility that EP_1_ and/or EP_3_ receptors are involved is diminished by the fact that transcripts for these receptors are very low in HFL-1 cells (GEO accession GSE73555). While several groups have reported that EP_2_ receptors do not internalize upon agonist exposure, we did not directly test this in our studies. Finally, our studies examined responses in a widely utilized cultured human fetal lung fibroblast cell line, and studies in primary pulmonary fibroblasts from humans as well as in vivo studies in animal models of lung fibrosis may shed new light on the unresolved issues from the present studies.

Given that cAMP has potent antifibrotic effects, any therapy addressing PF should consider cAMP-elevating agents to combat fibrosis. While PGE_2_ has long been an attractive candidate, its nonspecific activation of all EP receptors could activate unwanted signaling cascades. For this reason, EP_2_-specific agonists such as ONO-AE-259 should be considered due to their ability to only activate EP_2_-associated signaling pathways. However, EP_2_-specific agonists should likely be combined with PDE4-specific inhibitors so that cAMP elevation is promoted while simultaneously inhibiting EP_2_-mediated desensitization, thus achieving sustained high levels of cAMP that may be sufficient for reducing fibrotic activity. Selective and combinatorial inhibition of PDE isoforms is gaining traction in a wide array of pulmonary diseases (Zuo et al. 2019).

In conclusion, this study demonstrates that long-term exposure of lung fibroblasts to PGE_2_ induces agonist-specific desensitization of cAMP signaling without altering signaling by β-ARs. Selective agonists and antagonists demonstrate that the EP_2_ receptor subtype is responsible for both the cAMP signal stimulated by PGE_2_ and the subsequent desensitization. EP_2_ receptors do not undergo GRK-mediated phosphorylation and β-arrestin–mediated internalization, making this an unlikely mechanism for the observed desensitization. Increased PDE activity appears responsible for the desensitization, since PDE3A and PDE4D expression levels were selectively upregulated and bulk PDE activity was increased PGE_2_ pretreatment. The PDE4 inhibitor rolipram reversed the effect of PGE_2_ pretreatment. These findings provide potential new insights into PF pathology and point to new therapeutic approaches for treating PF.

## Figures and Tables

**Table 1:** Twenty-four-hour pretreatment with PGE_2_ desensitizes PGE_2_ responses in a concentration-dependent manner

Pretreatment condition	Log EC_50_
Vehicle	− 7.40 ±0.16
1 nM PGE_2_	− 7.10 ± 0.25
10 nM PGE_2_	− 6.79 ± 0.20
100 nM PGE_2_	− 6.09 ±0.15

HFL-1 cells were treated with vehicle and 1 nM, 10 nM, or 100 nM PGE_2_ for 24 h and washed, then acute cAMP responses to PGE_2_ were measured using the cADDis assay. Various concentrations of PGE_2_ ranging from 0.1 to 10 μM were added and responses measured as the change in fluorescence normalized to the change in fluorescence of maximal response stimulated by 1 μM forskolin plus 200 μM IBMX. The log EC_50_ was then calculated for PGE_2_ in each pretreatment condition. Data are mean ± SEM of *n* = 3–10

**Table 2:** EP_2_ receptors mediate PGE_2_ responses both before and after desensitization

Pretreatment condition	Antagonist	Log EC_50_
Vehicle	None	− 7.39 ±0.26
Vehicle	100 nM PF-0441894	− 6.44 ± 0.40
Vehicle	100 nM GW-627368X	− 7.92 ±0.24
100 nM PGE_2_ pretreatment	None	− 6.65 ± 0.27
100 nM PGE_2_ pretreatment	100 nM PF-0441894	− 5.68 ± 1.27
100 nMPGE2 pretreatment	100 nMGW-627368X	− 6.55 ± 0.37

HFL-1 cells were treated with vehicle or 100 nM PGE_2_ for 24 h, washed, then incubated for 10 min with either 100 nM PF-0441894 (EP_2_ receptor antagonist) or 100 nM GW-627368X (EP_4_ receptor antagonist). cAMP responses to various concentrations of PGE_2_ were measured using the cADDis assay. For each concentration of PGE_2_, the change in fluorescence was normalized to the change in fluorescence of maximal response stimulated by 1 μM forskolin plus 200 μM IBMX. The log EC_50_ was then calculated for PGE_2_ in each pretreatment condition. Data are mean ± SEM of *n* = 3–10. Plots of these data are shown in Fig. 4

## Abbreviations

AC: Adenylyl cyclase
β-AR: Beta-adrenoceptor
cAMP: Cyclic 3′,5′-adenosine monophosphate
Epac: Exchange protein activated by cAMP
EP_2_R: Prostaglandin E_2_ receptor
GPCR: G protein–coupled receptor
PDE: Phosphodiesterase PF Pulmonary fibrosis
PGE_2_: Prostaglandin
E_2_ PKA: Protein kinase A
qRT-PCR: Quantitative reverse transcription polymerase chain reaction
